# Calendar

**DOI:** 10.1155/EDR.2001.80

**Published:** 2001


					80
CALENDAR
April 27-28, 2001
6th Scientific Meeting of the
Hypertension in Diabetes
Edinburgh, Scotland, UK
Contact: Dr. John R. Petrie
Phone: 01 41 211 5412
Fax: 01 41 211 0414
e-mail:
j.r.petrie@clinmed.gla.ac.uk
May 25-28, 2001
Hypoglycaemia 2001: From
Research to Practice
Assisi, Italy
Contact: Lorena Briziarelli,
Organizing Secretariat
Di.M.I.
Via E. dal Pozzo
06126 Perugia, Italy
Phone: /39 (0)339 7299579
Fax: /39 075573 0855
e-mail:
lbriziar@dimisem.med.unipg.it
May 26-30, 2001
14th International Congress of
Comparative Endocrinology
Sorrento (Napoli), Italy
Contact: International
Federation of Comparative
Endocrinology, Studiocongressi,
Cicala de Pertis, Via S. Anna
dei Lombardi 38, Napoli, Italy
Phone: /39 081 551 1668
Fax: /39 081 552 8835
e-mail:
studiocongressi@napoli.com
June 9-13, 2001
5th European Congress of
Endocrinology
Turin, Italy
Contact: European Federation
of Endocrine Socieities, CCI
Centro Congressi
Internazionale, Maria Giovann
a Enria, Via Cervino, Torino,
Italy
Fax: /39 112446900
e-mail: efes2001@ibow.com
June 23-26, 2001
61st Annual Scientific Sessions
of the American Diabetes
Association
Philadelphia, PA, USA
Contact: American Diabetes
Association
1701 North Beauregard Street
Alexandria, VA 22311, USA
Phone: /1 703-549-1500
ext. 3553
e-mail: meetings@diabetes.org
July 21-22, 2001
Endocrinology and Diabetes
Bay Harbor, MI, USA
Phone: 800 800 0666 or
/1 734 763 1400
Fax: +1 734 936 1641
Please send conference and
symposium listings to:
628 N. 2nd Street
Philadelphia, PA, 19t23, USA
July 24-26, 2001
8th International Workshop on
Lessons from Animal Diabetes
Tokyo, Japan
Contact:
Yasunori Kanazawa, Chair
Abstract submission deadline:
April 30
Registration Secretariat:
c/o Access Brain Inc., Tokyo
Medical University, Shinjuku
6-1-1, Shinjuku-ku, Tokyo,
Japan 1.60-8402
Tel: /81 3 3351 6141
Fax: /81 3 3839 5035
e-mail:
pco2000@accessbrain.co.jp
website:
www.dm-net.co.jp/jaadr/lad8
September 9-13, 2001
(EASD) 37th Annual Meeting
of the European Association for
the Study of Diabetes
Glasgow, Scotland, UK
Contact: European Association
for the Study of Diabetes,
Merowingerstr. 29, D-40223
Dusseldorf, Germany
Phone: /49 211 316738
Fax: /49 211 316738
e-mail:
london@concorde-uk.com
September 20-22, 2001
Diabetes and the Heart
Nice, France
Contact: European Heart
House, Education Department
2035 Route des Colles
Les Templiers, BP 179, F-06903
Sophia Antipolis Cedex, France
Phone: /33 (0)4 92 94 76 00
Fax: /33 (0)4 92 94 18 24
e-mail: seminars@escardio.org
INTERNATIONAL JOURNAL OF EXPERIMENTAL DIABETES RESEARCH

				

